# [Retracted] Downregulation of microRNA‑195 promotes angiogenesis induced by cerebral infarction via targeting VEGFA

**DOI:** 10.3892/mmr.2023.13012

**Published:** 2023-05-15

**Authors:** Wen-Jing Zhao, Hai-Fang Zhang, Jin-Ying Su

Mol Med Rep 16: 5434–5440, 2017; DOI: 10.3892/mmr.2017.7230

Following the publication of this paper, it was drawn to the Editors’ attention by a concerned reader that the western blotting data shown in Fig. 3C were strikingly similar to data appearing in different form in another article by different authors at a diferent research institute. Owing to the fact that the contentious data in the above article were already under consideration for publication prior to its submission to *Molecular Medicine Reports*, the Editor has decided that this paper should be retracted from the Journal. The authors were asked for an explanation to account for these concerns, but the Editorial Office did not receive a reply. The Editor apologizes to the readership for any inconvenience caused.

